# Advances and Personalized Approaches in the Frontline Treatment of T-Cell Lymphomas

**DOI:** 10.3390/jpm12020267

**Published:** 2022-02-11

**Authors:** Mathew G. Angelos, Hatcher J. Ballard, Stefan K. Barta

**Affiliations:** Department of Medicine, Division of Hematology and Oncology, University of Pennsylvania, Philadelphia, PA 19104, USA; mathew.angelos@uphs.upenn.edu (M.G.A.); hatcher.ballard@pennmedicine.upenn.edu (H.J.B.)

**Keywords:** mature T- and NK-cell neoplasms, peripheral T-cell lymphoma, brentuximab vedotin, histone deacetylase inhibitors, lenalidomide, azacitidine, crizotinib, chimeric antigen receptor T-cell (CAR-T) therapy

## Abstract

Peripheral T-cell lymphomas (PTCLs) are a rare and heterogenous subset of non-Hodgkin lymphoma characterized by an aggressive clinical course. Historically, the treatment of PTCLs have been analogous to that of aggressive B-cell lymphomas; however, it has been well-established that overall responses and complete remission rates are far inferior using near-identical chemotherapy strategies. Recently, there has been a plethora of newer agents designed to target distinguishing cellular and molecular features of specific PTCL subtypes. These agents have been proven to yield superior anti-lymphoma responses and, in some cases, overall survival in the relapsed, refractory, and frontline treatment setting. In this review, we will summarize and highlight the most influential clinical trials leading to the Food and Drug Administration (FDA) approval of several novel therapeutic agents against PTCL, with an emphasis on emerging studies and strategies to expand their potential use in the frontline treatment setting.

## 1. Introduction

Peripheral T-cell lymphomas (PTCL) represent a spectrum of hematological diseases that account for 5–10% of non-Hodgkin Lymphoma (NHL) and 15–20% of aggressive lymphomas in Western countries [1]. PTCLs are a heterogenous collection of mature T- and NK-cell neoplasms that historically have been classified based on morphologic, immunophenotypic, and clinical features [2]. However, recent advancements in high-throughput genomic and molecular assays have identified unique mutational signatures, cellular origin, and cytokine profiles as crucial elements for diagnosis and subsequent novel targeted therapies [3,4,5,6]. This in turn led the World Health Organization (WHO) to recategorize mature T- and NK-cell neoplasms to include 27 distinct phenotypic entities in its newest revision of classification of lymphoid malignancies [7]. Despite these advancements in diagnostic granularity, roughly 30% of all PTCLs remain categorized as PTCL, not otherwise specified (PTCL-NOS), which further highlights ongoing disease complexity and challenges of implementing universal chemoimmunotherapies [1,8].

PTCLs can be broadly categorized as one of four clinical subtypes—cutaneous, extranodal, nodal, and leukemic. With the exception of most cutaneous T-cell lymphomas (CTCL), breast-implant associated anaplastic large cell lymphoma (ALCL), and T-cell large granular lymphocytic leukemia (T-LGL), the remaining PTCL subsets (the primary focus of this review) harbor aggressive clinical features and yield a generally overall poor prognosis. Induction therapies for both nodal and extranodal PTCLs have historically mirrored those of aggressive B-cell lymphomas, consisting of CHOP- (cyclophosphamide, doxorubicin, vincristine, and prednisone) or CHOEP- (CHOP + etoposide) based chemotherapy backbones. Early on, a single-institution, retrospective analysis demonstrated for 117 patients with PTCL-unspecified (PTCL-U, the most common histological subtype prior to the WHO designation of PTCL-NOS) receiving CHOP-based chemotherapy, an overall response rate (ORR) of 84% and a complete response (CR) in 64% of patients was observed; the 5-year progression-free survival (PFS) was only 29% and 5-year overall survival (OS) was 35% [9]. A retrospective, multivariate analysis of similarly treated patients with PTCL-U demonstrated worse OS to those stratified to higher risk groups (two or more adverse risk factors) based on the Prognostic Index for PTCL-U (PIT) score, calculated by age greater than 60, performance status, lactate dehydrogenase (LDH) level exceeding the laboratory upper limit of normal, and presence of bone marrow involvement [10].

The absence of durable responses led to two landmark prospective studies evaluating the role of high-dose chemotherapy with autologous stem cell transplantation (ASCT) following CHOP-based induction therapy in the upfront setting. In a seminal study, 83 patients (PTCL-NOS: *n* = 32 and angioimmunoblastic T-cell lymphoma (AITL): *n* = 27) underwent induction, with 66% of patients continuing to ASCT; the majority of remaining patients did not continue with ASCT due to progressive disease. With this approach, ORR was 79%, CR was 39%, and 3-year OS was 38%; however, this improved to 71% when stratified on patients who actually underwent transplant [11]. A larger, prospective phase II study enrolled 160 patients with PTCL who received either CHOP (if greater than 60 years of age) or CHOEP induction, followed by high-dose chemotherapy with ASCT. Here, 58% of patients continued with ASCT (16% had refractory disease and 26% had progressive disease or chemotherapy-induced toxicity). With this approach, the 5-year OS and 5-year PFS were 51% and 44%, respectively [12]. A population-based, historical outcomes analysis from the British Columbia Cancer Agency (BCCA) reported a median overall survival (mOS) of 5.5 months in those patients with refractory or relapsed PTCL who did not undergo ASCT [13]. Subsequently, following CR with induction therapies, high-dose chemotherapy followed by ASCT has become the de facto standard of care in transplant-eligible patients in the US, although randomized data evaluating this approach remain lacking.

Outcomes for patients for whom first line therapy failed, with the exception of ALCL, are even worse, with median PFS and OS ranging from only 3–4 months and 5–6 months, respectively, and less than 25% survival at three years post-relapse [13,14,15]. Given the dismal prognosis in the relapsed and refractory (R/R) setting, novel targeted and personalized therapies have been employed with variable degrees of efficaciousness in specific PTCL subtypes. In this review, we will highlight the clinical advancements of targeted and personalized therapy for PTCL, with a focus on PTCL-NOS, T- follicular helper phenotype lymphomas (i.e., angioimmunoblastic T-cell lymphoma (AITL)), and ALCL, and highlight those with the most potential to integrate into the frontline setting in the near future.

## 2. Novel Approaches to Frontline Therapies—Successes and Failures

### 2.1. Brentuximab Vedotin

Brentuximab vedotin (BV, previously referred to as SGN-35) is an antibody-drug conjugate (ADC) consisting of a chimeric anti-CD30 monoclonal antibody and the cytotoxic agent monomethyl auristatin E (MMAE) [16]. Mechanistically, BV binds to CD30, is internalized by clathrin-mediated endocytosis, and fuses with lysosomes causing the release of MMAE. MMAE induces cell cycle arrest and subsequent apoptotic cell death by inhibiting microtubule assembly and polymerization [16,17]. CD30 surface antigen is robustly expressed across a variety of PTCLs, with 43% of AITL, 64% of PTCL-NOS, 80% extranodal NK/T-cell lymphoma nasal type (ENTL), and virtually 100% of ALCL [18,19]. A landmark phase I, open-label, multicenter, dose-escalation study demonstrated efficacy of BV for patients with R/R CD30+ lymphomas. While 15 of the 17 patients enrolled had Hodgkin lymphoma, the two patients with CD30+ ALCL both achieved CRs following single-agent intravenous BV treatment at 1.8 mg/kg every 3 weeks [20]. These results paved way for a larger, phase II multicenter trial in which 58 patients with R/R CD30+ ALCL that were similarly treated with BV yielded an ORR of 86% and CR of 57%, resulting in FDA approval in 2011 [21]. An additional phase II study queried a more heterogeneous PTCL population, notably including a subset of patients (17%) with absent CD30 expression based on immunohistochemistry (PTCL-NOS: *n* = 21, AITL: *n* = 13). Patients with AITL fared better in terms of ORR, CR, and duration of response (54%, 38%, 6.74 months, respectively) as compared to PTCL-NOS (33%, 14%, 1.61 months, respectively) [22]. Intriguingly, significant reduction in tumor burden (upwards to ~75%) was observed in both AITL and PTCL subsets that did not express CD30, and there was no correlation between CD30 expression based on immunohistochemistry and response rates. These findings were in accordance with similar studies of single-agent BV and CTCL, where patients with low CD30 expression (<10%) based on immunohistochemistry were as likely to respond as those with higher CD30 expression [23].

Based on the relative efficacy in R/R PTCL, BV was investigated in the frontline setting. The initial phase I, single-arm study substituted vincristine for BV with the remainder of the CHOP-backbone (BV + CHP) in 26 patients with untreated CD30+ PTCL. While the majority of patients enrolled had ALCL (*n* = 19, 69%; ALK-negative: *n* = 16; ALK-positive: *n* = 3), the ORR was 100%, with 92% achieving CR. A total of 50% of patients remained in CR at the 5-year follow-up, and the mOS was not reached by this timepoint [24]. This provided the foundation for the ECHELON-2 study, a double-blinded, double-dummy, multicenter, phase III trial directly comparing BV + CHP every three weeks for 6–8 cycles (*n* = 226) head-to-head with CHOP every three weeks for 6–8 cycles (*n* = 226) in previously untreated PTCL with CD30+ expression >10%. Consolidative ASCT, allogenic stem cell transplantation (alloSCT), or radiotherapy was permitted after treatment based on investigator discretion. Again, the majority of patients enrolled were systemic ALCL (*n* = 316, ALK-negative: *n* = 218, ALK-positive: *n* = 98); however, PTCL-NOS (*n* = 72) and AITL (*n* = 54) were represented. From the 5-year updated intention to treat analyses, frontline treatment with BV + CHP yielded superior outcomes as compared to CHOP. At the median follow-up of 47.6 months, 5-year PFS was 51.4% with BV + CHP vs. 43.0% with CHOP, and 5-year overall survival (OS) rates were 70.1% with BV + CHP vs 61.0% with CHOP [25]. Comparable efficacy was also observed among patients who relapsed and subsequently received BV monotherapy, with an ORR of 59% in patients retreated following BV + CHP as compared to 50% following CHOP. Although ECHELON-2 was not powered to directly compare BV + CHP vs. CHOP among non-ALCL subgroups, responses trending towards superiority were observed in the major PTCL subtypes (despite wide confidence intervals) and were statistically significant in both ALK-positive and ALK-negative ALCL [25]. Adverse events (AEs) were generally similar between treatment arms, with peripheral sensory neuropathy reported in 52% of patients receiving BV + CHP and 55% of patients receiving CHOP. Cytopenias (predominantly neutropenia) were mitigated with the addition of G-CSF in the BV + CHP arm. Secondary malignancies were reported in 14 patients (BV + CHP: *n* = 6, CHOP: *n* = 8). Collectively, the findings derived from ECHELON-2 led to approval of BV + CHP for the frontline treatment of CD30+ PTCL, which has become a standard of care option, particulary for ALCL. Ongoing prospective studies are further investigating the efficacy of BV + CHP in the frontline setting for PTCL with CD30 expression <10% (clinicaltrial.gov ID NCT04569032) [26].

A retrospective analysis of patients with PTCL treated in studies of the German High-Grade Non-Hodgkin Lymphoma Study Group revealed that those younger than 60 years of age and with LDH levels less than the upper limit of normal per laboratory standard at the time of diagnosis had a 3-year event-free survival benefit when treated with CHOEP as opposed to CHOP in the frontline setting (75.4% vs. 51.0%, respectively) [27]. This led to a multicenter, phase II study to evaluate the addition of etoposide to BV + CHP (CHEP-BV), followed by BV consolidation in patients with newly diagnosed CD30-expressing PTCL. Preliminary results of 46 patients completing CHEP-BV from the 48 patients enrolled (AITL: *n* = 18, PTCL-NOS: 11, ALK-negative: 3, ALK-positive: 11) were significant for an ORR of 91% and CR of 81% at the completion of CHEP-BV. When stratified on CD30+ expression, the ORR and CR for patients with 1–9% CD30+ expression were 63% and 67%, respectively, and the ORR and CR for patients with >10% CD30+ expression were 93% and 67%, respectively. The 1-year PFS following completion of CHEP-BV was 82% in patients who ultimately received ASCT versus 48% in patients who did not receive ASCT [28]. These encouraging results demonstrate efficacy and feasibility of combination targeted and high-intensity chemotherapies in the frontline setting in those that are eligible across a diverse cohort of PTCL subsets.

### 2.2. Histone Deacetylase Inhibitors (HDACis)

Vorinostat, romidepsin, and belinostat comprise the HDACis with clinical activity in both CTCL and PTCL. The predominant mechanism of HDACis is interference with histone and chromatin modification, but they have also demonstrated activity in facilitating DNA damage, expressing tumor suppressing genes, and modulating apoptotic thresholds [29]. The use of HDACis for specific subsets of PTCL (specifically AITL) is of growing interest based on improvements in identifying conserved tumor-associated epigenetic signatures. Specifically, epigenetic regulators, such as TET2, IDH2, and DNMT3A, are recurrently mutated in 70%, 82%, and 29% of AITL, respectively, as well as in 49%, 0%, and 27% of PTCL-NOS, respectively [8,30,31].

Vorinostat initially demonstrated clinical efficacy in CTCL in two phase II studies, with an ORR of 24-30%, leading to its FDA approval in R/R CTCL [32,33,34]. A small phase I study of vorinostat in combination with CHOP for 6 cycles in untreated PTCL that enrolled 14 patients yielded a CR of 93% with a 2-year PFS and 2-year OS of 79% and 81%, respectively, and with a favorable toxicity profile [35]. While initially promising as a single agent additive to CHOP-based chemotherapy in the first line setting, combination with lenalidomide and dexamethasone yielded poor results, with significant dose-limiting toxicities [36]. More dedicated randomized studies may be able to parse out the definitive benefits of vorinostat in frontline and R/R PTCL and may be best served if stratified based on epigenetic signature profiles.

Romidepsin was also first identified to be effective in R/R CTCL prior to its investigation in the R/R PTCL setting. An initial open-label, phase II study of 131 patients with R/R PTCL (53% PTCL-NOS, 21% AITL, 16% ALK-negative ALCL) yielded an ORR of 25% and CR of 14.6% when treated with single-agent romidepsin. These responses were sustained at long-term follow-up with a median PFS (mPFS) of 29 months in initial responders and durable responses lasting greater than 12 months in 53% of these patients [37,38]. Following FDA approval in the R/R PTCL setting, a subsequent phase I/II study combining dose-reduced romidepsin (10 mg/m^2^ on Day 1 and Day 8 with CHOP × 6) was pursued in untreated PTCL. For the 37 patients (AITL: *n* = 15, PTCL-NOS: *n* = 9), response rates were favorable (for all-comers, the CR was 51% and the OS was 71%); however, despite dose reduction, there was a very high rate of hematological toxicity (Grade 3 or 4 thrombocytopenia: 78%, Grade 3 or 4 neutropenia: 89%) [39]. Based on these excellent responses and despite hematological toxicity, a randomized, phase III study (Ro-CHOP) was pursued comparing combination romidepsin + CHOP for six cycles versus CHOP for six cycles. Unfortunately, the combination romidepsin + CHOP did not result in superiority in either PFS or OS as compared to CHOP alone in all-comers, and again, hematological AEs led to either interruption of romidepsin (63%), CHOP, or ultimately discontinuation (8%) [40]. Notably, in an exploratory analysis, patients with an aggressive T-follicular helper cell (T_FH_; inclusive of AITL) PTCL-subset did have a modest PFS benefit with Ro-CHOP as compared to CHOP (19.5 months vs. 10.6 months), although this was not statistically powered to allow a definitive conclusion [41]. The recently reported PTCL13 phase I/II study investigated romidepsin + CHOEP followed by high-dose chemotherapy with either ASCT or allogenic stem cell transplant for the frontline treatment of PTCL. In a cohort of 86 patients (PTCL: *n* = 33, T_FH_ inclusive of AITL: *n* = 31, ALK-negative ALCL: *n* = 21, unclassifiable: *n* = 1) the 18-month PFS was 48%, which did not meet the planned statistical threshold for study continuation, and enrollment was stopped [42]. Unlike Ro-CHOP, there was no observed benefit specifically in the T_FH_ cell subgroup. Unfortunately, 24 patient deaths were recorded: 22 due to progressive lymphoma, one due to transplant-related mortality, and one due to a secondary malignancy. While the lack of efficacy may be related to a high proportion of patients with stage III or IV disease (91%), treatment-related toxicities continue to be a clear barrier in using romidepsin in the frontline with combination chemotherapy.

Belinostat demonstrated an ORR of 25% as monotherapy for a R/R PTCL-specific subgroup as a part of a phase II, open-label, single-arm, multicenter study that enrolled 53 patients (PTCL: *n* = 24, CTCL: *n* = 29) [43]. This led to a phase II study (BELIEF), in which 129 patients with R/R PTCL that received belinostat monotherapy (1000 mg/m^2^ intravenously for days 1–5 of a 21-day cycle) correlatively showed a 25.8% ORR, with an mOS of 7.9 months and mPFS of 1.6 months. Notably, when stratified on patients who achieved a CR, responses were durable, with the median duration of response not being met and exceeding 29 months at the time of publication [44]. Toxicity profiles were acceptable, with main Grade 3 or 4 AEs related to pneumonia (5.4%) and thrombocytopenia (2.3%). Following FDA approval in the R/R PTCL setting, belinostat was moved in combination with CHOP for six cycles in the frontline setting as a part of a phase I study (Bel-CHOP) that ultimately enrolled 23 patients after several years of recruitment. The ORR was 86%, with 71% of patients achieving CR and with the incidence of AEs similar to its equivalent use in the R/R setting [45]. Belinostat represents a potentially universal adjunct to standard CHOP chemotherapy in the frontline setting in non-ALCL PTCL and may potentially have less hematological toxicities than Ro-CHOP, pending assessment in a randomized trial.

### 2.3. Pralatrexate

Pralatrexate is a selective antifolate analogue that functions as a potent inhibitor of dihydrofolate reductase causing dysregulation of purine and pyrimidine synthesis in tumor cells [46]. Early clinical evaluation showed much greater activity in T-cell lymphomas than in B-cell lymphomas. In a seminal phase I study, four patients with PTCL achieved a CR, whereas 16 patients did not demonstrate CR or PR, for mechanistically unknown reasons [47]. This led to a phase II, single-arm, open-label, multicenter study (PROPEL), in which 115 enrolled patients with R/R PTCL received single-agent IV pralatrexate at 30 mg/m^2^ per week for 6 consecutive weeks in 7-week cycles. The ORR was 29%, with a median PFS and OS of 3.5 and 14.5 months, respectively [48]. Similar to other antifolate therapies, such as methotrexate or pemetrexed, the most common AEs were cytopenias (Grade 3 or 4: 33% thrombocytopenia, 22% neutropenia) and mucositis (Grade 3 or 4: 22%). Given positive outcomes in the R/R PTCL setting, with minimal significant toxicities, a phase II, single-arm, multicenter, study was developed, moving pralatrexate into frontline treatment, alternating with CEOP (cyclophosphamide, etoposide, vincristine, and prednisone) chemotherapy. Of the 33 patients enrolled, 64% of patients had PTCL-NOS, 24% of patients had AITL, and 12% had ALCL, with 46% of total patients possessing high/intermediate or high risk stratification. While 52% of patients achieved CR, and the 2-year PFS and OS were 39% and 60% respectively, these results were not statistically superior to historical controls that had received CHOP alone [49]. Given similar adverse side effects with pralatrexate as seen in prior studies, notably exceeding that observed with BV + CHP therapy, pralatrexate has not been integrated as an additional agent with frontline CHOP-based chemotherapy backbone for PTCL.

### 2.4. Mogamulizumab

Mogamulizumab is a humanized monoclonal antibody with a defucosylated Fc region that targets and inhibits CC chemokine receptor 4 (CCR4). CCR4 is endogenously expressed on T-regulatory (Treg) cells and also upregulated on T-cell neoplasms, specifically in advanced cases with peripheral blood involvement [50,51]. A lack of Fc segment fucosylation has been demonstrated to enhance antibody-dependent cellular cytotoxicity (ADCC) as compared to fully fucosylated monoclonal antibodies [52]. Mogamulizumab was initially investigated as a phase I/II study that enrolled patients with both relapsed CCR4+ acute T-cell leukemia/lymphoma (ATLL), PTCL, and CTCL. An ORR of 31% (5 of 16 patients) was observed, with one patient developing grade 3 dose-limiting toxicities (rash and febrile neutropenia) and grade 4 neutropenia. Notably, one PTCL-NOS patient had a CR while the others had stable disease, with PFS greater than 110 days [53]. This was followed with a dedicated phase II multicenter study of 35 patients with CCR4+ R/R PTCL. The ORR (11.4%) was much lower for all-comers as compared to the previously described study, hypothesized to be in part due to the inclusion of patients with refractory disease for CHOP-based chemotherapy [54]. Mogamulizumab was explored in the upfront setting in combination with chemotherapy (modified LSG15 regimen) in a Japanese phase II study, which demonstrated improvement in the ORR in newly diagnosed ATLL patients, but resulted in no improvement in PFS or OS [55]. As compared to randomized and comparative phase III studies, such as MAVORIC, which demonstrated a prolonged mPFS of 7.7 months in CTCL patients with mogamulizumab, equally favorable results have not panned out involving patients with PTCL, and thus it has not been favored either in the R/R or frontline settings [56].

### 2.5. Alemtuzumab

Alemtuzumab (also referred to as CAMPATH-1H) is a humanized anti-CD52 monoclonal antibody that has been previously employed in treating acute graft-versus-host disease following alloSCT, advanced stage chronic lymphocytic leukemia, and R/R T-cell lymphomas. The CD52 antigen is ubiquitously expressed on non-malignant, mature B- and T-cell subsets and further enriched in all PTCL, with the notable exception of ALCL [57,58,59]. Predating the BV era, three phase II studies demonstrated the efficacy of alemtuzumab in the frontline setting [60,61,62]. Gallamini et al. demonstrated that the addition of subcutaneous alemtuzumab to a CHOP backbone for eight cycles yielded an impressive CR of 71%. However, grade 4 neutropenia, CMV reactivation, and major infections (inclusive of Creutzfeldt-Jakob virus reactivation, pulmonary invasive aspergillosis, and *Staphylococcal* sepsis) were all seen, despite anti-infective prophylaxis [60]. Kluin-Nelemans et al. reported outcomes using an intensified alemtuzumab + CHOP combination every other week for 8 cycles from the Dutch-Belgian Hemato-Oncology Group (HOVON-69) study. While CR/PR was also relatively high (90%), this again came at the cost of severe infection (60% grade 3–5) and cytotoxicity, including one patient developing an EBV-associated lymphoproliferative disease secondary to alemtuzumab-induced EBV reactivation [62]. Binder et al. reported the use of alemtuzumab in the frontline consolidative setting following CHO[E]P every 2 weeks, yielding CR in 58.5% of enrolled patients; however, once again, this was complicated by grade 3 and 4 infections, cytopenias, and one treatment-related death [61]. A recent phase III, randomized trial compared alemtuzumab + CHOP (A + CHOP) to CHOP, specifically in elderly patients (61–80 years old) with untreated PTCL who were deemed unfit to pursue consolidative ASCT. With CHOP dosed bi-weekly over six cycles as a chemotherapy backbone in both arms, CR was achieved in 60% of those receiving A + CHOP compared to 43% in those receiving CHOP alone. However, there were no statistically significant benefits in 3-year EFS (27% vs. 25%) or 3-year OS (37% vs. 56%), with the latter complicated again by increased infection and development of B-cell lymphoma [58]. Collectively, these AEs have restricted the use of alemtuzumab in the frontline setting.

### 2.6. Lenalidomide

Lenalidomide belongs to a class of immunomodulatory drugs (IMiDs) that primarily functions to enhance ubiquitin ligase activity and subsequent proteasomal degradation of disease-specific proteins. Lenalidomide has been proven to be an effective treatment in multiple myeloma, B-cell lymphomas (particularly mantle cell lymphoma), chronic lymphocytic leukemia, and del(5q) myelodysplastic syndrome. Similar to other hematological malignancies, lenalidomide monotherapy is not particularly effective against R/R PTCL secondary to low rates of durable response as compared to other novel agents. In a phase II study, 40 patients with either untreated PTCL who were not candidates for upfront chemotherapy (*n* = 8) or R/R PTCL (*n* = 32) and treated with lenalidomide monotherapy had an ORR of 26%, with only 8% achieving CR, and a PFS of approximately 4 months [63]. Given its relatively well-tolerated safety profile, lenalidomide has since been studied as combination therapy in the front line, similar to treatment approaches used in other hematological malignancies. A phase II study of 40 patients with newly diagnosed PTCL receiving lenalidomide + CHOEP resulted in an ORR of 69% with a CR of 48%; however, AEs (mainly hematological) led to a high discontinuation rate [64]. A similar study combining lenalidomide with CHOP specifically in elderly patients with AITL yielded similar results and did not reach the primary endpoint [65]. In response, induction chemotherapy has been replaced by other novel agents, notably in a recently reported phase II study investigating the combination of induction lenalidomide with romidepsin for elderly patients who are not candidates for intensive chemotherapy. Of 29 enrolled patients, the ORR was 75% and hematological toxicities were decreased. CR was achieved in 30% of patients, and their subsequent median duration of response was 14.3 months [66]. Studies such as these highlight the potential for chemotherapy-free induction with novel agent combinations; randomized trials involving triplet modalities are currently enrolling.

### 2.7. Azacitidine

Azacitidine is a DNA hypomethylating agent that is theorized to be effective against PTCL, given its propensity for genomic alterations in epigenetic regulators, as previously described [67]. Azacitidine has already been integrated as standard of care therapy for certain myelodysplastic syndromes and as induction and maintenance therapy for acute myeloid leukemia [68]. In a retrospective series of 12 patients with AITL who received subcutaneous azacitidine for either concurrent myeloid neoplasm or as compassionate use therapy for R/R AITL, the ORR was 75%, with a CR of 50% [69]. This lead to a phase II, multicenter study of oral azacitidine (CC-486) + CHOP in the first line setting for PTCL. Notably, unlike other studies to date, the majority of the 21 enrolled patients were PTCL-T_FH_ (81%), with 14% PTCL-NOS, and 5% ATLL. The results were encouraging, with an ORR and CR after three cycles of 85% and 55%, respectively, and end of treatment ORR and CR of 76.5% and 76.5%, respectively. The 1-year PFS and OS were 69.9% and 93.8%, respectively, with the expected hematological toxicities observed [70]. This promising, upfront, combination approach is further being explored in parallel with a PI3K tyrosine-kinase inhibitor (duvelisib) as a part of the ALLIANCE/Intergroup A051902 study involving CD30-negative PTCL (clinicaltrial.gov ID NCT04803201).

### 2.8. PI3K Inhibitors (PI3Ki)

The phosphoinositide 3-kinase (PI3K) signaling network is an important downstream effector pathway of B- and T-cell receptor activation that drives clonal proliferation and differentiation [71]. For hematological malignancies, there are currently four PI3Kis that are FDA approved exclusively for use in R/R B-cell neoplasms: idelalisib, copanlisib, umbralisib, and duvelisib. Of these, duvelisib has shown the most promise to date for use against T-cell neoplasms. Duvelisib, also known as IPI-145, specifically inhibits two PI3K isoforms, PI3Kδ and PI3Kγ, which are constitutively expressed and required for robust T-cell receptor-dependent signaling in malignant T-cells. PTCL (*n* = 16) was included as a subgroup in IPI-145-02, a phase I, open-label, dose-escalation study of duvelisib in patients with advanced hematological malignancies. The ORR and CR were 50% and 19%, respectively, albeit some patients analyzed were enrolled in the dose escalation phase; the maximum tolerated dose was 75 mg twice daily (77% of patients) [72]. The most common grade 3 and 4 AEs were transaminitis (up to 40%), maculopapular rash (17%), and neutropenia (17%). Driven by encouraging in vitro efficacy data, combination duvelisib and romidepsin against R/R PTCL in a recently reported Phase I study yielded an ORR and CR of 58% and 42%, respectively, with an mPFS of 6.8 months [73]. Notably, combination therapy with duvelisib 75 mg twice daily and romidepsin 10 mg/m^2^ reduced the proportion of patients with Grade 3 and 4 transaminitis (14%), although there was increased neutropenia (36%). To improve on the AEs seen with duvelisib, the expansion phase of the phase II PRIMO trial allowed patients to receive duvelisib monotherapy at 75 mg twice daily for two cycles to maximize tumor control, followed by 25 mg twice daily as maintenance therapy. This strategy yielded an ORR of 50% and CR rate of 32%, with improved rates of grade 3 or 4 transaminitis (24.4%) [74]. As previously mentioned, the potential benefit of adding duvelisib to CHOP or CHOEP will be explored as a part of the ALLIANCE/Intergroup A051902 study for previously untreated CD30-negative PTCL.

### 2.9. Consolidative Stem Cell Transplantation

**Figure 1:**
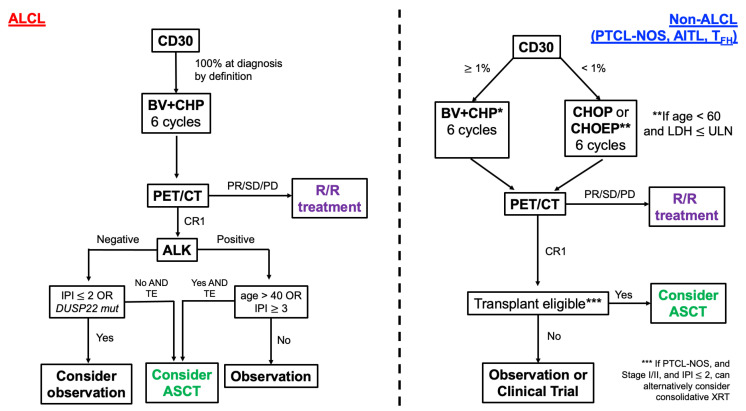
Suggested algorithm for frontline treatment of anaplastic large cell lymphoma (ALCL) and the most common non-ALCL peripheral T-cell lymphoma (PTCL) subsets. CD30 expression should be determined based on tissue biopsy immunohistochemistry analysis. LDH = lactate dehydrogenase, ULN = upper limit of normal, BV + CHP = brentuximab vedotin, cyclophosphamide, doxorubicin, prednisone, CHOP = cyclophosphamide, doxorubicin, vincristine, prednisone, CHO(E)P = cyclophosphamide, doxorubicin, vincristine, etoposide, prednisone, PET/CT = positron emission tomography/computed tomography, PR = partial response, SD = stable disease, PD = progressive disease, CR1 = complete response #1, IPI = international prognostic index, ASCT = autologous stem cell transplant, R/R = relapsed and/or refractory, TE = transplant eligible, XRT = radiotherapy, ALK, DUSP22. * While the authors favor BV + CHP for patients with CD30 expression ≥ 1%, CHOP or CHOEP for non-ALCL PTCL can be considered.

The role of consolidative stem cell transplantation as standard of care in fit patients with PTCL has come into question based on two recent analyses. Early ASCT following first remission has been historically associated with improved PFS (but not OS) for those with diffuse, aggressive, and high-intermediate or high risk NHL based on the results of the SWOG 9704 intergroup trial. Notably, this analysis combined both aggressive B- and T-cell lymphomas, with only 40 of 370 induction-eligible patients harboring an aggressive T-cell phenotype [75]. Furthermore, 30% of these patients were excluded from the study prior to randomization. A retrospective, subgroup analysis of T-NHL from SWOG 9704 compared outcomes of the 15 patients that continued to ASCT versus the 13 patients that were randomized to the control group, consisting of three additional cycles of chemoimmunotherapy without ASCT. While sample sizes were small, intriguingly there were no statistically significant differences in outcome (5-year PFS: 40% vs. 38% for ASCT vs. control, respectively; OS: 40% vs. 45% for ASCT vs. control, respectively) [76]. This was followed by a randomized, phase III clinical study directly comparing ASCT and alloSCT in 104 transplant-eligible patients with PTCL (except ALK-positive ALCL) following CR with four cycles of CHOEP induction chemotherapy. Interestingly, there were no statistically significant differences in 3-year EFS (43% vs. 38%) or OS (57% vs. 70%) between alloSCT and ASCT. In fact, in the alloSCT arm, none of the patients experienced disease relapse; however, eight patients (31%) ultimately died from treatment-related mortality, closing the study. Collectively, these data suggest no current role for alloSCT in consolidating a first remission of nodal PTCL, although it is strongly considered for certain highly aggressive and rare non-nodal TCL subtypes, such as hepatosplenic T-cell lymphoma (HSTCL) and ATLL. While the role of ASCT is still debatable, our current practice is to offer ASCT in transplant-eligible patients (1) with non-ALCL treated with BV + CHP or CHO(E)P, (2) with high-risk IPI ALK-negative ALCL, or (3) who are older and who have ALK-positive ALCL, based on outcome data according to prognostic scoring and genetic subtype (Figure 1) [77,78]. As an example, in considering specific molecular features, *DUSP22*-rearranged ALCL has been shown to have excellent prognosis, similar to ALK-positive ALCL, possibly rendering ASCT unnecessary, although this will have to be confirmed in larger series [79].

## 3. Emerging Personalized Therapies

### 3.1. ALK Inhibitors

Crizotinib is a prototypic example of a targeted therapy utilized successfully in treating R/R ALK-positive ALCL. Crizotinib is an oral, small molecule competitive inhibitor of ALK and MET kinase activity. In ALK-positive solid and hematological tumors, activating mutations in the tyrosine kinase domain of the ALK oncogene are considered driver mutations. Crizotinib has already demonstrated high response rates with minimal toxicity for the treatment of ALK-positive non-small cell lung cancer (NSCLC) and has already been established as standard of care first line therapy in lieu of platinum based chemotherapy [80]. The safety and efficacy of crizotinib was established from a phase I, pediatric consortium study that enrolled 79 children, a subset of which had R/R ALK -positive ALCL (*n* = 9, 36%). In this subgroup, the ORR was 88% and CR was 78%; the main Grade 3 and 4 AE was neutropenia (15% of total patients) [81]. This was followed in-tandem by a phase I study in adults with ALK-positive NHL (ALK-positive ALCL: *n* = 9, ALK-positive diffuse large B-cell lymphoma: *n* = 2) who received crizotinib monotherapy. Preliminary results are impressive, with an ORR of 91% (ORR of 100% in ALK-positive ALCL), including a CR of 82%. Longer term follow-up data demonstrated a median duration of response of approximately 10 months and a 2-year PFS of 63.7% [82]. Similar results were observed with alectinib, a second-generation ALK inhibitor [83]. Frontline studies are ongoing to assess ALK inhibitor efficacy in ALK-positive PTCLs as compared to chemotherapy, similar to the approach used in NSCLC patients (clinicaltrial.gov ID NCT01979536).

### 3.2. Cellular Therapy

CARs are fundamentally composed of the intracellular signaling domain from the endogenous T-cell receptor (TCR) linked to a single chain variable fragment (scFv) that functions as an antigen recognition domain. The scFv sequence is engineered from monoclonal antibody variable heavy (V_H_) and variable light (V_L_) domains via a short peptide linker. Novel generation CARs are further engineered with a costimulatory domain (either CD28 or 4-1BB) with or without additional “armor”, such as cytokine inducer sequences designed to enhance CAR-T trafficking, activation, or cytotoxic activity. Initial reports of safety and efficacy of CAR-T directed against B-cell malignancies were published approximately a decade ago. Subsequent studies investigating the use of CAR-T in heavily pre-treated R/R aggressive B-cell lymphomas demonstrated high rates of durable remission, even after prior salvage chemotherapy with stem cell rescue [84]. CAR-T therapy has since been expanded into treatment of other classes of hematological neoplasms, such as multiple myeloma, and has further been integrated as a standard of care option for chemotherapy-refractory patients [85]. Thus, CAR-T offers a theoretical option for chemotherapy-refractory and aggressive PTCLs in the future.

**Figure 2:**
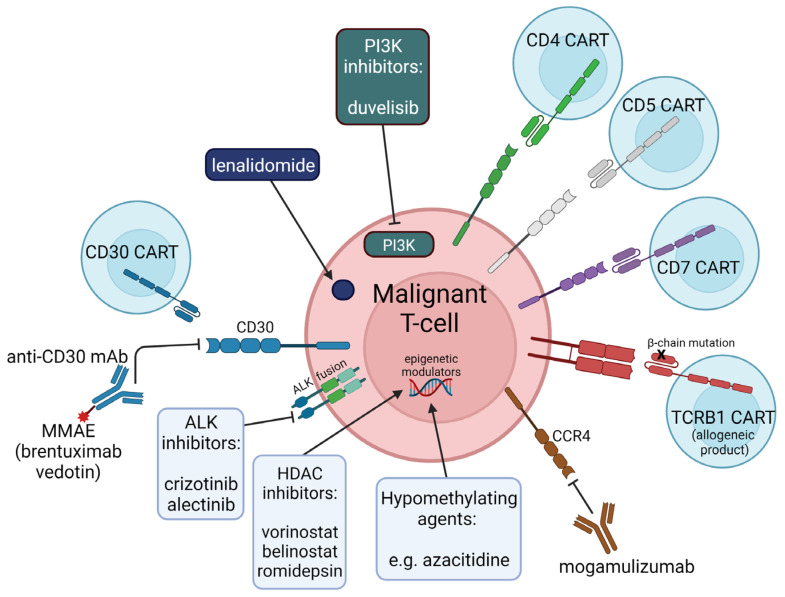
Schematic of novel biologic agents currently employed and potential future cellular therapies under development for clinical use against peripheral T-cell lymphomas. CAR = Chimeric antigen receptor, CART = CAR-T cell, MMAE = monomethyl auristatin E, HDAC = histone deacetylase, PI3K = phosphoinositide 3-kinase, TCRB1 = T-cell receptor beta chain 1, CCR4 = CC chemokine receptor 4.

Utilizing CAR-T against T-cell neoplasms has been challenging, primarily due to three adverse consequences: (1) fratricide, (2) severe T-cell aplasia, and (3) product contamination. CAR-T fratricide, due to endogenous expression of T-cell antigens that CARs are designed against, limits CAR-T expansion and manufacturing. Preclinical and early phase I clinical studies are investigating gene editing of the CAR antigen target in CAR-T to bypass fratricide. T-cell aplasia, unlike B-cell aplasia, has the inherent risk of permanent immunosuppression and predisposition to life-threatening infection, which to date is the major obstacle in translating T-neoplasm directed CAR-T therapy to the clinic. One possibility, however, is employing CAR-T solely as a “bridge to transplant”, such that high-dose conditioning chemotherapy prior to an autologous stem cell rescue ablates the residual CAR-T and promotes reconstitution of a complete immune compartment. Lastly, product contamination, in which neoplastic T-cells are inadvertently engineered into CAR-T, leads to the theoretical risk of developing CAR-refractory disease. One potential in circumnavigation is through the use of allogenic T-cells (with inactivating mutations in the endogenous T-cell receptor to prevent graft-versus-host responses) or the use of alternative cytotoxic cell sources (such as CAR-NK-cells or CAR-macrophages). A summary of CAR-T antigen targets currently under investigation in early phase clinical trials are highlighted in Figure 2. In inherently chemotherapy-refractory and heterogenous diseases such as PTCL, employing cellular therapy early on may have the potential to overcome the issues leading to poor outcomes that have notably remained unchanged for the majority of patients.

## 4. Conclusions

PTCLs are a complex and heterogenous subset of mature T- and NK-cell neoplasms in which defined categorization and subsequent therapies are continuing to evolve. Improvements to genomic and molecular diagnostic methods have identified key cellular features that have informed novel targeted therapeutic use and have improved overall outcomes. Over the last two decades, the armamentarium against aggressive PTCLs has expanded from anthracycline-based chemotherapy followed by ASCT to now include FDA-approved monoclonal antibody therapy (brentuximab vedotin), HDACis (romidepsin and belinostat), folate antimetabolites (pralatrexate), and crizotinib. On the horizon, possible future approvals include targeted therapies specific to distinct molecular subsets, such as azacitidine, duvelisib, and lenalidomide. CAR-T therapy further represents a novel and, to date, a largely understudied possibility for patients that are refractory or non-responsive to chemotherapy. Optimistically, the number of actively enrolling clinical trials for novel therapies against PTCLs continues to increase, many of which have harnessed unique biological identifiers with personalized and targeted approaches (Table 1). The results of these therapies in the R/R setting will be crucial to identify agents that can be translated to the frontline setting and hopefully provide efficacious and chemotherapy-free treatment.

## Figures and Tables

**Table 1:** Summary of key, actively recruiting, U.S.-based clinical trials for patients with peripheral T-cell lymphoma (PCTL). * = Untreated PTCLs eligible for frontline enrollment, BV = brentuximab vedotin, ASCT = autologous stem cell transplant, CHP = cyclophosphamide, doxorubicin, prednisone, ALCL = anaplastic large cell lymphoma, CHO(E)P = cyclophosphamide, doxorubicin, vincristine, etoposide, prednisone, R/R = relapsed and/or refractory, ATLL = Acute T-cell leukemia/lymphoma, CHEP = cyclophosphamide, doxorubicin, etoposide, prednisone, EPOCH = etoposide, prednisone, vincristine, cyclophosphamide, doxorubicin, CAR-T = chimeric antigen receptor T-cell, TRBC1 = T-cell receptor β-chain constant region 1.

Trial Number	Sponsor	Experimental Treatment	Phase	Status	PTCL Status
NCT04334174 *	Univ. of Kansas	BV after ASCT	II	Recruiting	BV + CHP induction in CD30+ PTCL
NCT03719105 *	New York Medical College	Pralatrexate + BV + chemotherapy	I	Recruiting	PTCL (non-ALCL or non-NK leukemia/lymphoma)
NCT01716806 *	Seagen, Inc.	BV	II	Recruiting	CD30+ PTCL
NCT04569032 *	Seagen, Inc.	BV + CHP	II	Recruiting	CD30+ PTCL < 10%
NCT04803201 *	Alliance for Clinical Trials in Oncology	Duvelisib or azacitidine (CC-486) + CHO(E)P	II	Recruiting	CD30+ PTCL < 10%
NCT03728972 *	MSKCC	Pembrolizumab	II	Recruiting	NK/T-cell lymphoma
NCT04639843 *	National Cancer Institute (NCI)	Doxorubicin + azacitidine + romidepsin + duvelisib	I	Not yet recruiting	New and R/R PTCL
NCT02737046 *	University of Miami	Belinostat + zidovudine	II	Recruiting	ATLL
NCT03264131 *	UNC	BV-CHEP	II	Recruiting	ATLL
NCT04301076 *	National Cancer Institute	Lenalidomide + EPOCH	I	Recruiting	ATLL
NCT04795869	Northwestern University	BV + pembrolizumab	II	Not yet recruiting	R/R PTCL
NCT04747236	Univ. of Virginia	Azacitidine + romidepsin	II	Recruiting	R/R PTCL
NCT03240211	Univ. of Virginia	Pembrolizumab + Decitabine and/or Pralatrexate	I	Recruiting	R/R PTCL
NCT03598998	City of Hope	Pralatrexate + pembrolizumab	I/II	Recruiting	R/R PTCL
NCT03534180	City of Hope	Romidepsin + venetoclax	II	Recruiting	R/R PTCL
NCT03278782	M.D. Anderson	Romidepsin + pembrolizumab	I/II	Recruiting	R/R PTCL
NCT03011814	City of Hope	Durvalumab +/− lenalidomide	I/II	Recruiting	R/R PTCL
NCT04447027	National Cancer Institute	Romidepsin + azacitidine + dexamethasone + lenalidomide	I	Recruiting	R/R PTCL
NCT04703192	Daiichi Sankyo, Inc.	Valemetostat	II	Recruiting	R/R PTCL
Cellular Therapy for R/R PTCL					
NCT04712864	Legend Biotech USA, Inc.	CD4 CAR-T	I	Recruiting	R/R CD4+ PTCL
NCT03690011	Baylor College of Medicine	CD7 CAR-T	I	Recruiting	R/R CD7+ PTCL
NCT04984356	Wugen, Inc.	CD7 CAR-T	I	Recruiting	R/R CD7+ PTCL
NCT04004637	PersonGen BioTherapeutics	CD7 CAR-T	I	Recruiting	NK/T-cell lymphoma
NCT04083495	UNC	CD30 CAR-T	II	Recruiting	R/R CD30+ PTCL
NCT04526834	Tessa Therapeutics	CD30 CAR-T	I	Recruiting	R/R CD30+ PTCL
NCT02917083	Baylor College of Medicine	CD30 CAR-T	I	Recruiting	R/R CD30+ PTCL
NCT04136275	Massachusetts General Hospital	CD37 CAR-T	I	Recruiting	R/R CD37+ PTCL
NCT03590574	Autolus Limited	TRBC1 CAR-T	I/II	Recruiting	R/R TRBC1+ PTCL

## Data Availability

Not applicable.
